# Advancements in integrated robotic sensing: A European perspective

**DOI:** 10.12688/openreseurope.16918.2

**Published:** 2024-07-02

**Authors:** Carmelo Mineo

**Affiliations:** 1 Institute for High-Performance Computing and Networking (ICAR), National Research Council, Palermo, 90146, Italy

**Keywords:** Robotics; Robotic NDT; Sensing Technologies; Structural Integrity; European Research

## Abstract

Robotic Non-destructive Testing and Sensing stands at the forefront of technological innovation, offering capabilities in assessing structural integrity, safety, and material quality across diverse industries. This comprehensive review article provides a detailed exploration of the field, focusing on the substantial contributions of European researchers and institutions. The need for non-destructive testing has been a constant in industries that rely on structural integrity, including aerospace, manufacturing, energy, construction, and healthcare. Traditional testing methods, such as radiography, ultrasonic testing, magnetic particle testing, and dye penetrant testing, have been integral for quality control and safety assurance. However, the robotisation of such methods has marked a profound shift, enabling precise, fast, efficient, and repeatable testing while minimising human exposure to hazardous environments. European researchers and institutions have played an instrumental role in driving the evolution of integrated robotic sensing. The historical perspective of the field reveals the pioneering spirit of Europe, as collaborative initiatives led to the development of robotic platforms equipped with advanced sensors and testing techniques. A critical aspect of the European impact on robotic inspection applications lies in developing advanced sensors, innovative robotic platforms, novel robotic path-planning and control approaches and data collection and visualisation tools. These developments continue to influence the global landscape of robotic sensing. European researchers remain at the forefront of current trends and innovations as the field continues to evolve. This review article will delve into these recent advancements, highlighting Europe’s pivotal role in pushing the boundaries of technology and application. The implications and applications of robotic sensing reverberate across multiple sectors worldwide. From inspecting critical aerospace components to ensuring the quality of manufactured goods, these technologies underpin safety and quality standards.

## Introduction

**Figure 1.**
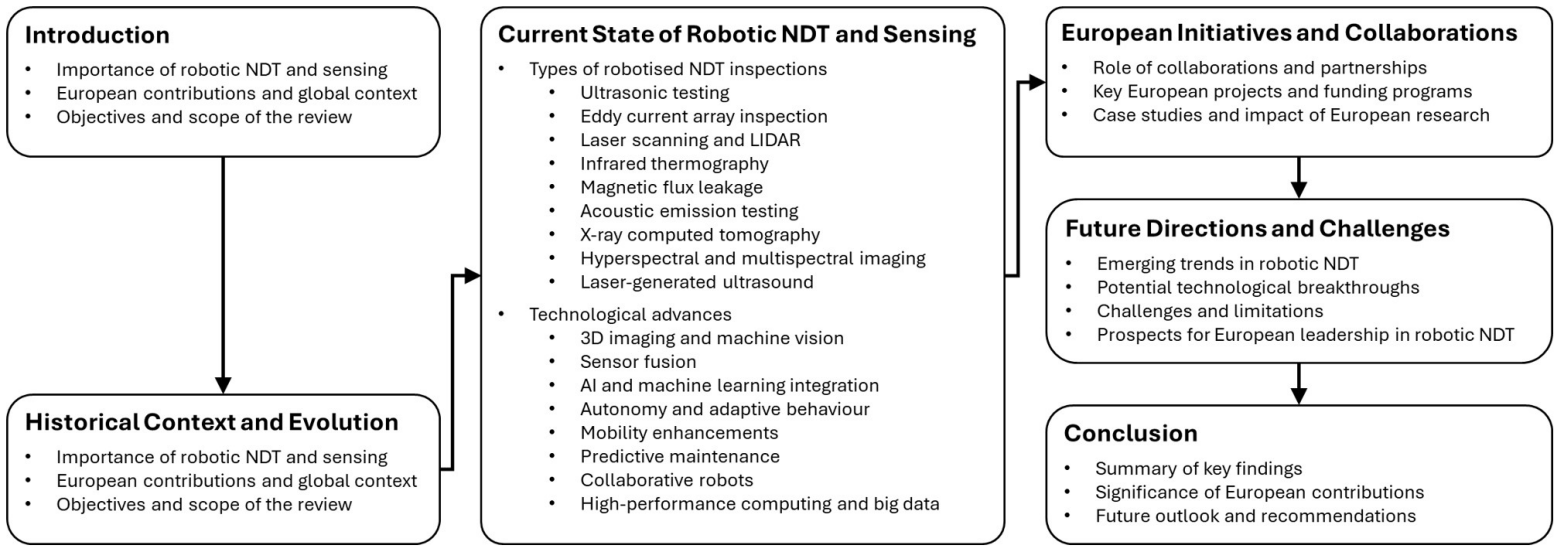
Flowchart illustrating the structure and key themes of this review article.

In the contemporary industrial fabric, robotic Non-Destructive Testing (NDT) and robotic sensing are not just novel adjuncts but foundational elements for ensuring the integrity, safety, and longevity of critical infrastructures and complex machinery. This technology stands at the forefront of the industrial maintenance and inspection revolution, offering unprecedented precision, efficiency, and safety. The importance of robotic NDT and sensing in maintaining the robustness of industrial applications cannot be overstated, especially in sectors where the cost of failure is measured not only in economic loss but also in human and environmental terms ^1, 2^. Against this backdrop, this review article meticulously canvases the European contributions to the evolution and application of robotic NDT and robotic sensing technologies. It aims to map the contours of progress in this domain, situating the European efforts within the global narrative of technological advancement. The scope of this review is deliberately broad. However, focused—it traverses across the deployment of robotic NDT and sensing in diverse industrial contexts, such as aviation, renewable energy, construction, and offshore operations, while concurrently highlighting the synergy between technological trends and the European Union (EU)’s strategic directives. The objectives of this review are threefold: firstly, to consolidate the significance and advancements of robotic NDT and sensing as they pertain to contemporary industrial applications; secondly, to present a critical assessment of the European projects and collaborations that are at the pinnacle of this technological wave; and thirdly, to prognosticate the future developments and potential paradigm shifts that these technologies may engender within the European industrial landscape. This review was constructed upon a foundation of comprehensive literature and project surveys by adopting a rigorous methodology. Searches were executed across a spectrum of scientific databases, including but not limited to Scopus, Web of Science, IEEE Xplore, and the EU’s COmmunity Research and Development Information Service (CORDIS) repository. A deliberate choice of keywords, encompassing phrases such as “robotic NDT innovation”, “European robotic sensing advancements”, “automation in industrial inspection”, and “EC-funded robotic projects”, served to filter the vast sea of information available. Review materials were selected by stringent criteria tailored to distil the most impactful and current contributions. Peer-reviewed articles and comprehensive technical reports demonstrating significant advancement or application in robotic NDT and sensing were prioritised. A particular emphasis was placed on extracting findings from projects that have received the European Commission’s endorsement or funding, reflecting the review’s commitment to spotlighting endeavours symbiotically linked to the EU’s research and innovation frameworks. Works developed in the European region, which did not receive direct European Commission funding, were also included. Most recent references have been preferred. The selection process eschewed materials that did not meet the specified criteria, such as those that lacked empirical data, did not directly engage with the core technologies of interest or were not in alignment with the research and innovation standards upheld by European directives. The resultant compilation of literature and projects sheds light on the technological triumphs and critically examines the sustainability, scalability, and socioeconomic impact of robotic NDT and sensing within the European context. In drafting this article, the review also adopted a meta-analytic approach to literature, discerning patterns and extracting insights from the convergence of multiple studies and reports. This robust approach ensures that the review is not merely a repository of disparate facts but a cohesive and critical narrative that presents a state-of-the-art understanding of robotic NDT and sensing technologies, with an eye firmly on the horizon of future possibilities. By charting this course, the review is a testament to Europe’s position at the vanguard of industrial inspection and maintenance technologies, highlighting the region’s commitment to innovation, safety, and excellence. The narrative offers a reflective look at the past, a nuanced examination of the present, and a speculative gaze into the future of robotic NDT and sensing technologies framed within the dynamic and collaborative spirit that characterises European research and technological development. For the sake of helping the reader, Figure 1 provides a flowchart that outlines the structure and key themes of this review article.

## Historical context and evolution

The inception of robotic NDT and robotic sensing can be traced back to the industrial automation surge of the late 20th century. In the early days, robotic systems were rudimentary and predominantly operated in large-scale manufacturing environments, such as automotive assembly lines, where they performed repetitive tasks with little variation ^3^. The evolution of robotics within NDT began as an offshoot of these developments, aiming to address the growing need to ensure product quality and structural integrity without causing damage to the items under scrutiny. Europe has been at the forefront of this evolution, with contributions spanning from fundamental research to the development of industrial applications. The journey commenced with simple automated systems designed to handle and position parts for inspection by stationary sensors ^4^. The turning point was the advent of microcomputers and advancements in sensor technology in the 1980s ^5, 6^, which enabled the development of more sophisticated and versatile robotic systems. The introduction of the EU’s Framework Programmes for Research and Technological Development in the 1980s ^7^ substantially boosted collaborative research and innovation in robotic NDT. These programs facilitated cross-border partnerships between academia and industry, fostering a fertile ground for technological advances in the field. Notably, the early emphasis was on enhancing the precision and reliability of robotic systems, which was crucial for the adoption of NDT in safety-critical sectors such as aerospace and nuclear energy. By the 1990s, the integration of robotics with non-destructive evaluation (NDE) technologies had garnered significant interest ^8^. The Framework Programmes continued to play a pivotal role, with projects increasingly focusing on incorporating intelligent sensing mechanisms into robotic systems. This period saw the emergence of robotic NDT systems capable of adapting to different materials and geometries, an advancement that was propelled by parallel progress in areas such as ultrasonics ^9^, radiography ^10, 11^, and eddy current testing ^12^. The turn of the millennium marked the beginning of a digital revolution within the field. The European Commission’s continued support through initiatives such as Horizon 2020 paved the way to integrating sensing technologies with industrial robot manipulators ^13– 15^. These advancements allowed for the automation of data collection ^16, 17^. The last decade has witnessed the harmonious convergence of robotics, NDT, and cyber-physical systems, underlined by European projects that emphasised the role of robotics in the Fourth Industrial Revolution or Industry 4.0 ^18^. Improved NDT data analysis and visualisation have significantly improved detection accuracy, defect characterisation, and decision-making processes ^19, 20^. The trend towards smart factories, equipped with interconnected robotic systems capable of self-diagnosis and autonomous operation has become increasingly prevalent. European research has been integral to these developments, with a notable example being the contribution to the field of adaptive robotics, where systems self-optimise to dynamically changing conditions ^1, 21^. In retrospect, the European contribution to robotic NDT and sensing has been characterised by consistent innovation, cross-disciplinary integration, and strategic collaboration. As we stand on the cusp of the next era of technological breakthroughs, the historical evolution within Europe provides a testament to the region’s pioneering spirit and sets the stage for the future trajectory of robotic NDT and sensing technologies.

## Current state of robotic NDT and sensing

### Types of robotised NDT inspections

The efficacy of robotic NDT hinges on the application of advanced sensing techniques. These techniques have evolved to address the growing complexity of industrial needs, ensuring precision and reliability in detecting and characterising potential defects. Nowadays, the sophistication of robotic sensing techniques represents a symbiosis of advanced sensor technology and robotic delivery systems. As these techniques are refined and integrated, robotic NDT sets new benchmarks for inspection accuracy, reliability, and thoroughness across various industrial applications. Ultrasonic testing (UT) has seen significant enhancements through robotics. Automated systems now utilise phased array ultrasonic testing (PAUT) ^16^ and time-of-flight diffraction (TOFD) ^22^ techniques, which allow for more detailed imaging and accurate defect sizing. When deployed via robotic arms or crawlers, these advanced UT methods can cover large areas and adapt to complex surfaces, which is essential in sectors such as aerospace. Robotic systems have also harnessed the power of eddy current array (ECA) inspection to detect surface and near-surface flaws in conductive materials. ECA sensors integrated with robotic platforms enable the inspection of large surface areas with higher speeds and improved detection capabilities compared to traditional point-by-point eddy current probes ^23^. Robotic NDT platforms have adopted laser scanning technologies and Light Detection and Ranging (LIDAR) systems to perform dimensional inspections, corrosion mapping, and defect detection ^24, 25^. The high-resolution data obtained from these laser-based techniques are invaluable for the geometric inspection of complexly shaped components and for creating accurate 3D models for further analysis. Infrared thermography has been integrated into robotic NDT to detect subsurface defects through thermal imaging ^26^. Robots equipped with thermal cameras can automate the scanning of large structures, such as composite aircraft parts, for heat signatures indicative of delamination or water ingress. Robotic systems can use magnetic flux leakage (MFL) technology to inspect ferromagnetic materials. This method is especially prevalent in the inspection of pipelines and tank floors, where robotic mobile platforms (crawlers) can systematically scan and provide real-time data on corrosion and pitting ^27^. Acoustic emission (AE) testing is one of the first inspection techniques the industry adopted. In robotic NDT, AE has been used for in-process NDT inspection (e.g. in robotic polishing ^28^). Mobile robots with inspection capabilities are also equipped with AE sensors and are being developed to enable autonomous navigation in complex and hazardous environments ^29^. Recent advancements in robotic handling of radiography equipment, particularly X-ray computed tomography (CT), allow for non-intrusive inspection of components with complex internal geometries ^30^. This method provides high-resolution cross-sectional images, facilitating the detection of internal defects without disassembling the part. Emerging in robotic NDT is hyperspectral and multispectral imaging, which captures data across multiple wavelengths to identify materials and detect anomalies that are not visible to traditional cameras or the naked eye ^31^. Another emerging technology used for robotic NDT is based on laser-generated ultrasound (LUT), which do not need any physical contact with the part to inspect and is attractive for remote inspection in hazardous environments ^32^.

### Current technological advances

The current state-of-the-art robotic NDT systems reflect a significant shift from manual inspection methods to automated, autonomous, high-precision, and efficient processes that substantially benefit safety, cost, and data quality. Modern robotic NDT systems increasingly adopt sophisticated technologies such as 3D imaging, machine vision, and advanced ultrasonic methods, facilitating accurate defect detection and measurement in complex geometries. Robotic NDT systems are increasingly equipped with various sensors that complement each other. For instance, the fusion of visual sensors with acoustic and ultrasonic sensors has enabled the detection of subsurface flaws that are not discernible to the naked eye or traditional imaging ^33^. Visual sensors, such as cameras and optical scanners, provide high-resolution images of the surface and near-surface regions. When combined with acoustic or ultrasonic sensors, which penetrate deeper into the material, visual sensors help create a more comprehensive understanding of the inspected object. The data from multiple sensors can be synthesised using advanced algorithms, providing a more comprehensive analysis of the material or structure under examination. The desire to integrate multiple sensor modalities within a single robotic platform focuses on developing appropriate methods to fuse multisensory data, which can lead to enhanced fault characterisation and material property evaluation capabilities. Despite the recent works ^34, 35^, multisource data fusion is still an open problem ^36^. One of the cornerstones of the current European landscape in robotic NDT is the integration of Artificial Intelligence (AI) and Machine Learning (ML) algorithms ^37– 40^. Soft computing techniques, including fuzzy logic, genetic algorithms, and neural networks, are increasingly utilised in NDE for tasks such as signal processing, pattern recognition, and decision making. These methods offer robustness and flexibility, accommodating the uncertainties and variabilities inherent in NDE data. AI and ML algorithms are deployed to automate the analysis of complex NDE data, identifying patterns and anomalies that may be imperceptible to human inspectors. For example, convolutional neural networks (CNNs) and support vector machines (SVMs) are used for image-based inspection tasks, such as defect detection in welds and composite materials. Deep learning, a subset of ML, leverages multi-layered neural networks to model intricate relationships within data. In NDE, DL techniques are employed for tasks such as ultrasonic signal classification, radiographic image interpretation, and predictive maintenance. These approaches improve detection accuracy and reduce false positives, enabling the interpretation of vast datasets with improved accuracy and the ability to learn from historical inspection data to optimise future inspections, thereby enhancing the reliability of NDE processes. To ensure the necessity and quality of research in this area, various standards and codes are being established. Organisations such as the American Society for Non-destructive Testing (ASNT), the International Organization for Standardization (ISO), and the European Federation for Non-Destructive Testing (EFNDT) are providing guidelines and standards for the implementation of AI, ML, and DL in NDE. These standards ensure that new technologies are rigorously tested and validated before being applied in industrial settings ^41^. Technological advances are converging towards autonomous, efficient, intelligent, and integrated systems with their operational ecosystems ^18^. These innovations signify a substantial leap forward in the capabilities of robotic NDT, with significant implications for safety, cost savings, and operational efficiency across multiple sectors.

Autonomy in robotic systems has transitioned from basic path-following to adaptive behaviour in complex environments ^1, 42, 43^. Advanced navigation systems, often powered by simultaneous localisation and mapping (SLAM) algorithms, allow robots to understand and navigate their surroundings in real-time ^44– 48^. That is particularly beneficial for inspecting intricate structures or navigating hazardous or confined spaces where human operation would be risky or impractical. Robotic platforms have seen vast improvements in mobility, enabling access to previously unreachable areas. From magnetic crawlers that can traverse vertical steel surfaces to drones that can inspect high-rise structures, the enhanced mobility of these robots expands the scope and efficiency of NDT tasks. Implementing machine learning algorithms in robotic NDT systems is expected to enable predictive maintenance capabilities. Such systems will be not only able to identify defects but also to predict their growth and suggest optimal maintenance schedules. The adoption of these predictive analytics aims to reduce downtime and extend the lifespan of components and infrastructure. Another distinct mention has to be done for the rise of collaborative robots (cobots), designed to work alongside human operators, combining the precision and reliability of robotic systems with the decision-making capabilities of humans. In the NDT field, cobots are expected to assist humans in a variety of settings, from manufacturing floors to in-field inspections, ensuring safety and improving inspection workflows ^49, 50^. Finally, advances in high-performance computing aim to enable the handling of large volumes of data generated during robotic NDT operations. The ability to process and analyse this ‘big data’ in near-real time has significant implications for the speed and accuracy of inspections, as well as for the development of digital twins that can simulate and predict the behaviour of physical inspection systems ^18^.

## European initiatives

### Role of collaborations and partnerships

Europe has seen the rise of robotic NDT across various sectors. In the aerospace industry, robotic systems equipped with advanced sensors ensure the integrity of critical components, adhering to stringent quality standards. The automotive industry utilises these systems for quality assurance in manufacturing processes, from casting to assembly line inspection. Energy sectors, including nuclear and renewables, are increasingly relying on robotic NDT to ensure the longevity and safety of their infrastructure. Robotics as a Service (RaaS) models are emerging in the NDT sector, where inspection services are offered through a subscription rather than direct robot purchase ^51^. This model allows rapid scalability and access to the latest technologies without significant upfront capital investment.

The current European research and development in robotic NDT is significantly influenced by collaborative projects and initiatives, often supported by the European Commission’s past and present funding schemes, such as Horizon Europe. In line with the principles of Industry 4.0, European industries are embracing smart factories where robotic NDT systems communicate with other digital systems within the manufacturing environment ^52^. This connectivity allows for real-time quality control and process optimisation. The robotic NDT and robotic sensing field in Europe is a testament to the power of collaboration and partnership across academia, industry, and government: these multi-sector networks pool resources, expertise, and infrastructure to drive innovation and technological advancements. Collaborative networks and partnerships are the backbone of progress in the European robotic NDT and sensing domain. They promote innovation and ensure that developments are aligned with market needs and regulatory standards. As European initiatives continue to encourage such collaborations, it is expected that the impact will be seen not only in technological advancements but also in Europe’s competitive position in the global NDT industry. The success of these collaborations also serves as a model for how various sectors can synergise to accelerate technological and commercial growth in other fields.

The European Institute of Innovation and Technology (EIT) functions as an autonomous entity within the EU, possessing legal status. Its establishment dates back to 2008, with the primary objective of enhancing Europe's innovation capabilities ^53^. The EIT operates through three fundamental pillars of activity, encompassing entrepreneurial education programs and courses conducted throughout Europe to mold students into entrepreneurs, provision of business creation and acceleration services to scale ideas and nascent businesses, and the undertaking of innovation-driven research projects. These projects facilitate the transformation of conceptual ideas into tangible products by fostering connections among partners, investors, and experts. Embedded within the European Union's Framework Program for Research and Innovation known as "Horizon Europe" ^54^, specifically under Pillar 3, denoted as "Innovative Europe", the EIT actively contributes to realising the Horizon Europe Strategic Plan's four crucial strategic orientations. These include championing an open strategic autonomy by spearheading the development of critical digital, enabling, and emerging technologies, sectors, and value chains; rejuvenating Europe's ecosystems and biodiversity while sustainably managing natural resources; positioning Europe as the forefront of a digitally-enabled circular, climate-neutral, and sustainable economy; and cultivating a more resilient, inclusive, and democratic European society. The EIT assumes a pivotal role in promoting innovation within manufacturing, encompassing areas such as robotic NDT. Through the collaboration of eminent organisations and research institutes, EIT Manufacturing expedites the advancement of cutting-edge technologies, seamlessly integrating them into the manufacturing sector.

EuRobotics is a Brussels-based international non-profit association for all stakeholders in European robotics. It builds upon the success of the European Robotics Technology Platform (EUROP) and the academic network of EURON, leading towards establishing one sustainable organisation for the entire European robotics community. The association was nurtured by a coordination action funded by the EC under FP7, which started in 2010 and ended in December 2012. EuRobotics was founded in September 2012 to strengthen Europe’s competitiveness and ensure the industrial leadership of manufacturers, providers, and end-users of robotics technology-based systems and services. As a prime networking organisation, euRobotics has been instrumental in shaping the robotics community’s direction ^55^. It promotes collaboration between stakeholders in European robotics research, development, and deployment. In the context of robotic NDT, euRobotics acts as a catalyst for initiating and nurturing partnerships that drive innovation.

The EU has endorsed Public-Private Partnerships (PPPs) to leverage the strengths of both sectors and support research projects in advanced robotics, including those specific to NDT applications. Since 2014, euRobotics has collaborated with the European Commission in the Public-Private Partnership SPARC under Horizon2020 to develop and implement a strategy and a roadmap for research, technological development, and innovation in robotics ^56^. SPARC has now ended, and a new partnership was created (ADRA – AI, Data and Robotics Association) in May 2021, with euRobotics as a founding partner ^57^. Through ADRA, euRobotics is actively collaborating with the Horizon Europe programme. The Clean Sky Joint Undertaking has been another PPP funded by the European Commission, which has had the two most extensive research programmes for aviation ever launched in Europe: Clean Sky (2008–2017, under FP7) and Clean Sky 2 (2014–2024, under Horizon 2020) ^58^. These programmes have been set up to accelerate the development of cleaner air transport technologies for the earliest possible deployment and have funded several projects related to adopting robotically enabled NDT in the aerospace sector. PPPs have enabled sharing high-cost R&D infrastructure, combining industrial insight with academic research rigour, which is essential for the expensive and complex development of robotic NDT systems. They help standardise robotic NDT technologies, facilitate the exchange of best practices, and enhance the European workforce’s skill set to meet future needs. The creation of consortia also means that research findings and innovations are more rapidly transferred from laboratory to market, significantly shortening the development cycles for new robotic NDT solutions. The collaborations enabled by funded initiatives allow industrial representatives, academics, and researchers to create valuable professional networks. As a result, the benefits of funded projects and partnerships go beyond the end of such ventures. Many research institutes exploit their members’ networks by inviting key people to form a steering group of experts. For example, the Fraunhofer Institute for Non-Destructive Testing IZFP has a Board of Trustees, which advises the institute’s management on essential issues, promoting and extending the relations to organisations interested in the institute’s research.

### The scale of EU-funded initiatives

The CORDIS database was interrogated using a Boolean search, combining multiple chosen search terms using precise logical relationships, such as AND and OR. This search approach was used to obtain precise and relevant search results by specifying the relationships among the search terms, saving time and effort while minimising the likelihood of encountering irrelevant or unrelated material. The following Boolean search string was used: *“(‘robot’ OR ‘robotic’ OR ‘robotically’ OR ‘roboti?ed’) AND (‘non-destructive’ OR ‘inspection’ OR ‘evaluation’ OR ‘NDT’ OR ‘NDE’ OR ‘sensing’ OR ‘sensor’)*”. This resulted in searching projects whose title and short description (teaser) contained at least one of the words in the first set of brackets and at least one in the second set. Note that the “ *?”* in *‘roboti?ed’* allowed looking for the presence of both the British English word “robotised” and the respective American English version “robotized”.

Additionally, the search results were filtered according to the funding schemes. To review the recent landscape, only projects funded through the HORIZON 2020 and HORIZON EUROPE schemes were considered. The described filtered search returned 1371 projects. The following metadata was extracted from the CORDIS repository for each of the found projects: the project start date, the end date, the total cost, the total EU contribution, the fields of science related to the project, the coordinating institution, and the participating institutions. The fields of science of each project are given as a list of strings detailing the fields of science related to the project. Each string shows a variable-depth hierarchy from the broadest classification to specific fields (e.g., “engineering and technology/materials engineering/composites”), following the hierarchical framework adopted by the European Commission ^59^. Finally, whereas each project has one and only one coordinator, it can have none, one or multiple participants. For the coordinator and each participant (if present), the following information was extracted: country of the coordinating/participating institution, amount of EU contribution received and amount of other funds available to the institution.

**Figure 2.**
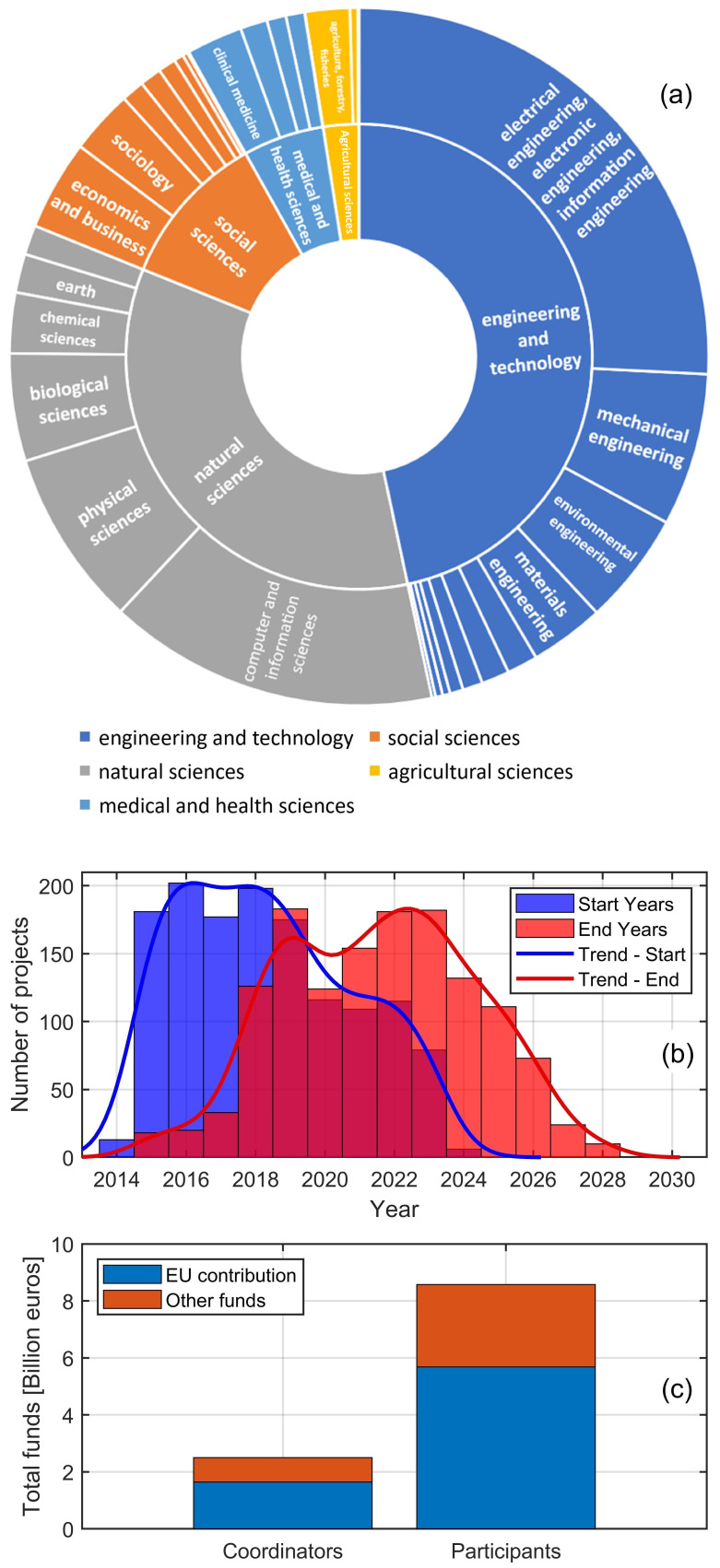
Sunburst chart of the fields of science **(a)**; Distribution of project start and end years **(b)**; Total project funds available to coordinators and participants **(c)**.

Thus, the project metadata extracted from CORDIS was thoroughly analysed. The sunburst chart in Figure 2a offers a lucid overview of the diverse scientific disciplines of the selected projects. The analysis of the fields of science strings has revealed a hierarchical depth going up to the seventh classification level, showing great permeance of robotic NDT and robotic sensing into numerous and specific fields. The distribution of projects across a wide range of science categories highlights the current priorities in the field and provides a quantitative insight into the European Commission’s strategic funding directions. To present a readable illustration, the sunburst chart shows the distribution of the detected fields of science only up to the second-level classification. Such a chart, though simplified, effectively captures the expansive essence of the field under review. It illustrates how diverse disciplines are intricately woven together to advance the capabilities of robotics in non-destructive testing and inspection. This convergence of disciplines into research projects is a testament to the complexity of the challenges at hand and a reflection of the collaborative and interdisciplinary approach necessary to address them. The sunburst chart, therefore, serves as both a visual summary and a quantitative analysis of the state-of-the-art in robotic non-destructive testing and inspection.

The found projects are well related to the following five broadest classification fields: Engineering and Technology (46.6%), Natural Sciences (34.5%), Social Sciences (10.8%), Medical and Health Sciences (5.6%), and Agricultural Sciences (2.5%). Within the broad category of ‘Engineering and Technology’, a notable emphasis is seen in ‘Electrical Engineering, Electronic Engineering, and Information Engineering’, which takes 55.3% of the broad field, reflecting the critical role of electronics and information technology in advancing robotic capabilities. That is followed by ‘Mechanical Engineering’ (15.2%), indicating significant research activity in robot design, movement, and operation mechanics. ‘Materials Engineering’ accounts for 7.4%, underscoring the importance of material science in developing advanced, durable, and efficient robotic systems and innovative materials. Here, the data show a noticeable concentration of research activities in areas such as ‘Composites’, highlighting the industry’s focus on developing advanced materials. Intriguing sub-sectors like ‘Carbon Fibers’, ‘Biocomposites’ and ‘Coating and Films’ demonstrate the field’s commitment to cutting-edge technology, sustainability, inspection, and maintenance. Other fields, such as ‘Environmental Engineering’ and ‘Civil Engineering’ (11.2% and 2.8% of the broad field, respectively), reveal the interdisciplinary nature of the research. Civil engineering projects likely focus on applying robotics in infrastructure inspection, while environmental engineering projects may explore using robots in assessing and preserving environmental health. While ‘Engineering and Technology’ is a major pillar, the presence of ‘Natural Sciences’, particularly ‘Computer and Information Sciences’ (44.4% of the broad field), underscores the pivotal role of computing and informatics in modern robotic systems. This field’s prominence indicates a heavy reliance on advanced algorithms, data processing, and software development in robotic inspection systems. ‘Social Sciences’, with ‘Economics and Business’ as its predominant sub-category (38.8% of the broad field), reflect an intriguing dimension. This suggests a keen interest in the economic impacts, business models, and market dynamics surrounding the deployment of robotic technologies in various industries. ‘Sociology’ represents 27.5% of the broad field, which makes it the second major sub-category of ‘Social Sciences.’ The relationship between sociology and NDT requires further exploration. One of the key intersections between sociology and robotic NDT is the study of human-technology interaction. Sociologists investigate how individuals and organisations perceive, adopt, and integrate modern technologies, such as robotic NDT systems, into their practices. This research is essential for understanding the social dynamics that influence the successful implementation of these technologies in various industries. For instance, introducing robotic NDT might necessitate new training programs for workers, leading to shifts in job roles and skill requirements. The role of robotic NDT in ensuring the safety and reliability of critical infrastructures is another area of interest for sociologists. Research in this field explores how different communities perceive the risks associated with infrastructure and the role of robotic NDT in mitigating those risks. Such studies are crucial for developing effective public communication strategies and building trust in innovative technologies. For example, public perception of robotic inspections in the nuclear energy sector can influence policy decisions and industry practices. The ‘Medical and Health Sciences’ field, predominantly in ‘Clinical Medicine’ (46.2% of the broad field), indicates the application of robotic inspection techniques in healthcare, perhaps in diagnostics, surgical assistance, or patient care robotics. Integrating robotic technologies in medicine demonstrates a crossover of engineering prowess into life-saving applications. Lastly, the ‘Agricultural Sciences’, focusing on ‘Agriculture, Forestry, and Fisheries’ (82.9% of the broad field), shows the application of robotics in enhancing productivity and sustainability in these sectors. Robots could be used for crop monitoring, precision farming, or environmental assessment.

The distribution and predominance of these fields on the sunburst chart highlight the diversity of applications and the interconnectivity between different scientific disciplines. It reveals how advancements in one area, like computing or materials science, can propel innovations in others, such as medicine or agriculture. As illustrated in the chart, this multidisciplinary approach is essential in addressing today’s complex challenges. It demonstrates that the future of robotic non-destructive testing and inspection lies not just in technological advancements but also in integrating these technologies across various sectors of society.

The analysis of the input start and end dates reveals an average project duration of 3.4 years, with most projects having a duration of 3.5 years (median value). The standard deviation of the project duration distribution is 1.3 years, indicating that almost 70% of the projects have a duration between 2.1 and 4.8 years. The bar plot in Figure 2b provides a visual representation of the temporal dynamics of the analysed projects. It illustrates a peak in project initiations between 2016–2018, with a gradual decline observed afterwards. Conversely, project completions show a trend peaking in the current year (2023), indicating a maturation of the field. All of the analysed projects are expected to end by 2028. The distribution of project timelines, as evidenced by the provided plot, aligns with the Horizon 2020 and Horizon Europe programs.

**Figure 3.**
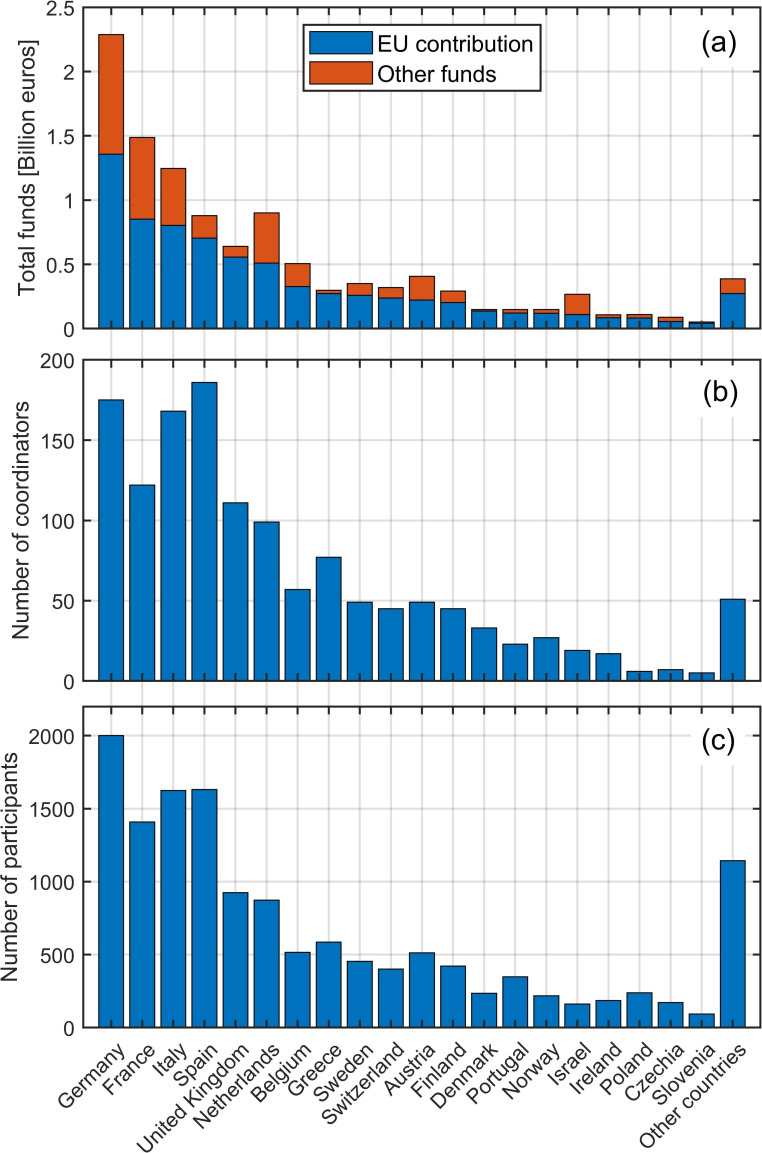
Total project funds for first 20 countries **(a)**; Total number of coordinating roles per country **(b)**; Total number of participating roles per country **(c)**.

The project’s concentration starts around the mid-2010s and corresponds with Horizon 2020’s launch in 2014, which marked an increased focus on integrating robotics into various industrial and societal applications. The anticipated completion of many projects towards the late 2020s reflects the transition into the Horizon Europe framework, which aims to build upon its predecessor’s successes and drive Europe’s scientific leadership forward. The overlap and continuity between these funding programs demonstrate the EU’s long-term approach to fostering cutting-edge research and innovation fields that involve robotic NDT and robotic sensing. Figure 2c focuses the attention on the total funds. The total cost of the selected projects amounts to over 11 billion euros, with a total EU contribution of 7.3 billion euros (66.2%). Thus, the percentage of other funds is 43.8% of the total. That has most likely been allocated by governmental and federal parties, which provides a clear picture of the support and prioritisation of robotic sensing research within the broader research landscape. The plot clearly illustrates the cumulative funds provided to the project coordinating institutions and the other participants. The disparity in total funds between coordinators and participants is due to the presence of more participants than coordinators. However, since the metadata of the considered projects reveals an average number of 10.3 participants per project, the average funds received by a project participant is about three times lower than the average funds available to a coordinator. That is coherent with the typical strategic role coordinators play in leading project direction and execution and is reflected in the substantial EU contribution coordinators receive. The co-funding mechanism present in most EU projects encourages a synergistic effect, leveraging additional resources to amplify the impact and reach of European research and innovation. Figure 3a shows the top 20 countries that received the highest total EU contribution, with Germany being the first country, followed by France, Italy, and Spain. The additional total funds (other funds) used by each country are generally comparable and proportional to the EU contribution, except for the Netherlands, Austria, and Israel, where the additional funds are proportionally higher than those available in the other countries. The additional funds may well reflect the internal policy of each country for funding science, research, and innovation.

With projects from Horizon 2020 concluding and Horizon Europe initiatives still ongoing, there is an emphasis on the practical application and impact of research, leading to advancements in technology and their implementation in real-world settings. Considering that many Horizon Europe proposals are still under evaluation and new calls are continually being opened, it is possible to anticipate several outcomes for the future of robotic non-destructive testing and inspection. The investigated historical dataset and the ongoing activities in Horizon Europe suggest a robust and dynamic future for this field. The continuation of funding opportunities indicates a sustained commitment to research and innovation in this field, suggesting that these technologies will continue to evolve and be a focus of European research agendas. There will likely be a fresh wave of project initiations, making the plot in Figure 2b outdated quite rapidly. Horizon Europe emphasises intersectoral and interdisciplinary research and encourages international cooperation, leading to more innovative robotic non-destructive testing projects that integrate different fields and technologies.

### Pivotal European projects

The remaining part of this section examines several pivotal European projects, representative of the extensive project database investigated so far, shedding light on their objectives, methodologies, and outcomes. These representative projects are described chronologically to help the reader grasp the progressive advancements in the field under review and the shift of interests in the scientific and industrial community. Between 2015 and 2017, the AEROWORKS project ^60^ developed an innovative aerial robotic team capable of autonomously conducting infrastructure inspection and maintenance tasks while providing human operators with an intuitive, user-friendly interface. AEROWORKS developed a new class of Unmanned Aerial Vehicles (UAVs). Dubbed ‘Collaborative Aerial Robotic Workers’, these UAVs were equipped with dexterous manipulators, novel physical interaction and co-manipulation control strategies, perception systems and planning intelligence.

SmokeBot ^61^ was primarily driven by application needs for robots that operate in domains with limited visibility. It focused on civil robots that support fire brigades in search and rescue missions, such as post-disaster management operations in response to tunnel fires. SmokeBot addressed this challenge by delivering software and hardware components that facilitate robot systems performing under harsh smoke, dust, or fog conditions. The project team achieved this through sensor fusion, where the outputs of the robot’s novel 3D radar camera, stereo thermal camera and gas sensors are integrated, interpreted, and used by the robot.

The AEROARMS project developed ^62^ intelligent aerial robotic manipulators, including arms and multi-thrust platforms (tilted rotors) that could exert forces in any direction. Thanks to its advanced AI, the drones could hold onto an object with one arm while inspecting it with another. Their capabilities were successfully demonstrated in real-life situations, including wall thickness measurements of pipes and tanks.

The CompInnova project ^63^ focused on developing an innovative inspection methodology with automated and manual capabilities for composite and metallic aircraft structures. The novel structural integrity approach comprised a qualified phased array and infrared thermography method attached to a mobile vortex robot, a damage tolerance structural integrity assessment technique processed on a computer and an innovative repair system.

AEROBI ^64^, driven by the bridge inspection industry, developed low-flying automated robots with arms, intelligent control, computer vision, and sensing. In case of concrete swelling or spalling, the aerial robots could establish contact with the bridge to measure the delamination and diameter of reinforcing steel bars non-destructively.

The ACCURATE ^65^ project was part of the Clean Sky 2 programme. Its goal was to obtain the optimum technology for the full non-destructive inspection of both present and future-generation hybrid-material aircraft and thick composite structures made of acoustic damping materials. This project demonstrated the inspection of complex parts through LUT by robotic delivery of the laser ultrasound excitation and detection pulses. A 6-axis lightweight robot arm equipped with flexible optical fibres was used. The robotic deployment of LUT enabled full non-contact inspection of large areas. Besides integrating the robotic system, ACCURATE developed bespoke signal processing algorithms to reduce coherent noise from optical fibres and enhance signal-to-noise ratio, using LUT synthetic aperture focusing.

The HYFLIERS project ^66^ unveiled robotic technologies to decrease the cost and risks of current human inspection in production plants. This project developed and tested the first aerial-ground robotic prototypes with a hyper-redundant, lightweight, articulated arm. The robotic arm was equipped with an inspection sensing subsystem and a ground support unit for efficient and safe inspection in industrial sites. Advanced automatic collision detection and avoidance algorithms enabled accurate guidance, positioning, landing, and rolling on constrained surfaces, such as pipes.

SheaRIOS ^67^ developed a remote-controlled, robotic solution for inspecting wind turbine blades (WTBs) that can be used while the turbine operates. The developed robotic crawler uses vacuum-assisted caterpillar tracks to manoeuvre to an area of interest, where the shearography inspection of the WTB is performed. The SheaRIOS system was remotely controlled from ground level using a high-bandwidth data link within an umbilical cable. The robotic climber acted as a distribution hub for power and communications for the crawler and the inspection unit. Using a robotic solution in the wind turbine sector not only means inspections can be done while a turbine is in operation but also means they can be done more safely, regularly, and affordably.

The goal of the ROBINS project ^68^ was to fill technology and regulatory gaps for adopting robotics and autonomous systems (RAS) in the life-cycle surveys of ships. The project was coordinated by the Italian Naval Register (RINA) and tested a variety of RAS. These included a collision-tolerant drone for inspecting irregular confined spaces like ballast tanks and a semi-autonomous drone for surveying bulkheads and structures inside large cargo holds. ROBINS also developed a crawler: a small, agile robotic vehicle for close-up surveys that can climb steps, manoeuvre around corners, and reach and probe structures. Furthermore, the project developed various innovative software tools for autonomous RAS inspections, leveraging emerging technologies such as LIDAR, photogrammetry, machine learning, artificial intelligence, and 3D model augmentation for navigation, localisation, and data acquisition.

SPIRIT ^69^ aimed to develop a framework for robots that took the step from programming of complex inspection tasks to configuring such tasks. The project results included an offline software module with features such as model-based automatic coverage planning for complex parts, automatic robot program generation and an inline module that could deal with sensor data mapping to transfer 2D measurements to the 3D object model.

The BugWright2 project ^70^ demonstrated an adaptable autonomous robotic solution for servicing the outer hulls of ships. It combined the survey capabilities of autonomous micro-air vehicles (MAV) and small autonomous underwater vehicles (AUV) with teams of magnetic-wheeled crawlers operating directly on the structure’s surface. The project aimed to facilitate a multi-robot visual and acoustic inspection, detecting corrosion patches or cleaning the surface as necessary. The developed technology is adaptable to inspecting and maintaining storage tanks or other structures assembled from metal plates.

The RIMA project ^71^ aimed to facilitate the uptake of inspection and maintenance technologies by establishing a network of 13 Digital Innovation Hubs on robotics. The project enabled the sharing of best practices and provided education and training on automated inspection and maintenance operations to accelerate growth in the field.

The ERABID project ^72^ was a H2020 Marie-Curie Individual Fellowship conducted by the author of the present review article. It proposed a new generation of robotic inspection approaches, moving from traditional automated NDT towards more autonomous NDT solutions. The scalability of the developed approaches makes them suitable for different scenarios for applications beyond automated non-destructive testing. For example, the autonomous geometry mapping of complex parts and autonomous robotic navigation are suitable for space exploration programs and land mapping through drones. This project outcome can be used to support digital and robotic innovation and can impact distant fields. Although this project did not release new products to the market, the executed trials can easily translate to prototypes and industrial impact.

The Robotics4EU project ^73^ aims to ensure a more widespread adoption of AI-based robots in healthcare, inspection and maintenance of infrastructure, agri-food, and agile production. The project’s core concept is that the technological research and innovation must be driven by society by considering its societal impact. Thus, Robotics4EU advocates for integrating non-technological aspects such as ethics, law, socioeconomics, data, privacy, and gender into technology development and involving the end-users, either citizens or professional users of robots. An emerging playground for advancing robotic inspection systems is the European decommissioning and dismantling (D&D) market for nuclear facilities, characterised by significant long-term growth.

The advancements in adaptive and autonomous inspection robotic systems have inspired the ongoing CLEANDEM project ^74^. It aims at developing a technological breakthrough for D&D operations that will save time, reduce costs, and minimise human intervention while increasing safety. The project objective is to inspect nuclear-contaminated environments by delivering a cyber-physical system consisting of an uncrewed ground vehicle platform equipped with innovative radiological sensing probes. Such a system will allow the creation of a 3D and fully detailed digital twin of the surveyed area augmented with radiological information. CLEANDEM is funded through the H2020 EURATOM programme, which aims to pursue nuclear research and training activities with an emphasis on continually improving nuclear safety, security and radiation protection, notably to contribute to the long-term decarbonisation of the energy system in a safe, efficient and secure way. While the EURATOM programme does not explicitly target robotic non-destructive testing and evaluation, specific funded projects, like CLEANDEM, are very relevant for advancing the field.

The “edge air” project ^75^ wants to revolutionise the field of autonomous inspection robots by using advanced on-the-edge AI technologies. The idea is based on a robot-agnostic AI-driven software platform capable of enabling a diverse fleet of mobile robots to conduct routine inspections autonomously in industrial plants, including remote and hazardous facilities. With semantic scene understanding, deeply connected data analysis and novel sense and react capabilities, such a platform shall provide artificial ‘common sense’ for the execution of inspections in unstructured and frequently changing industrial environments. The project can expand into new segments – mining, construction, security, and agriculture.

### Financial classification and impact of funded projects

**Figure 4.**
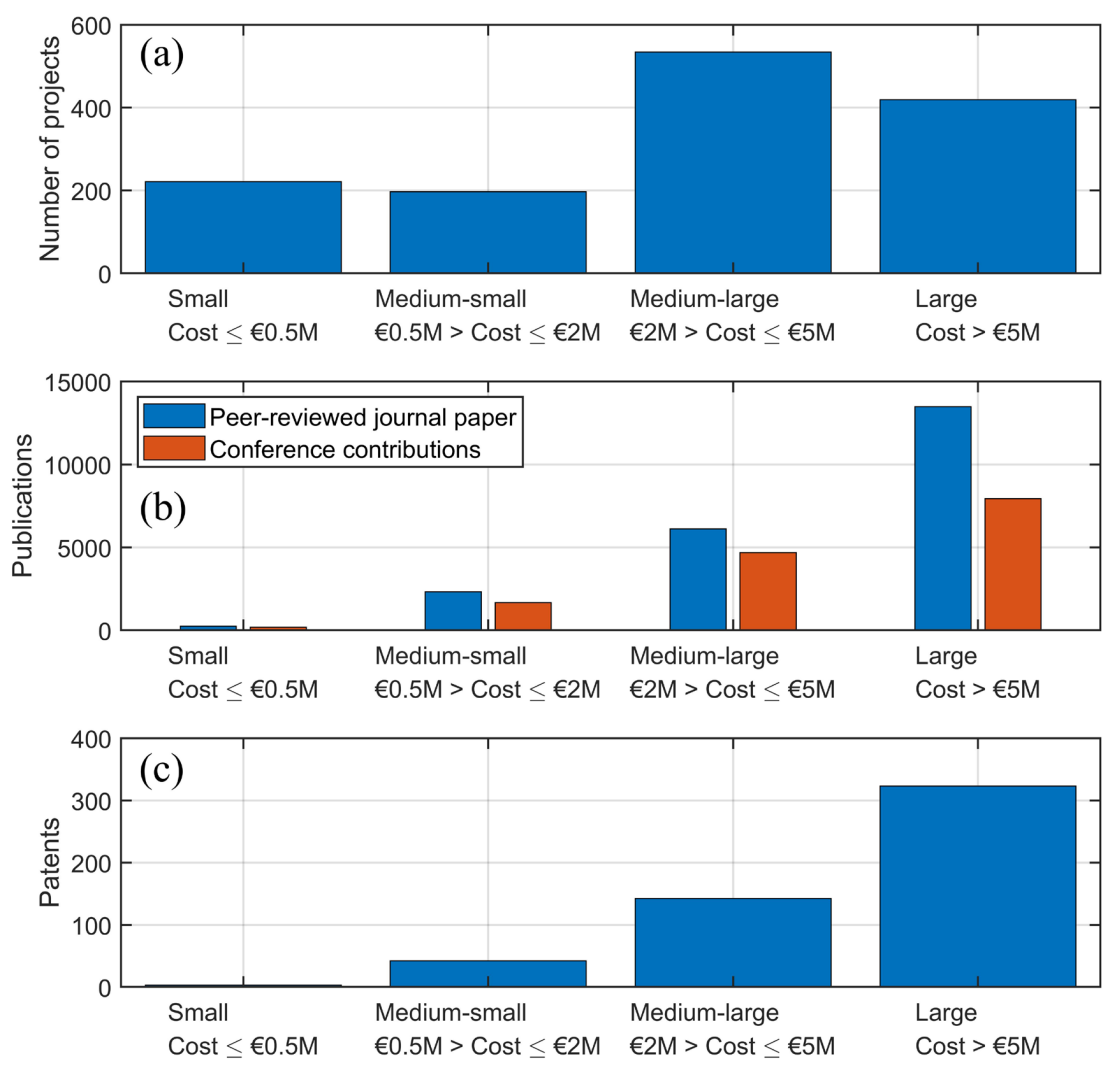
Distribution of funded projects in robotic sensing based on financial classification ( **a**), cumulative number of publications ( **b**) and number of patents ( **c**).

The financial scope of funded projects in robotic sensing varies significantly, reflecting the diverse nature and scale of these initiatives. Figure 4a classifies the projects into four categories based on their total budgets: small, medium-small, medium-large and large-scale.

Small-scale (up to €0.5M) projects typically focus on specific technical challenges or pilot studies. Despite their limited budgets, they have often played a crucial role in advancing niche technologies or validating new concepts. An example of a small-scale project is ERABID ^72^, which received under €0.2M for advancing autonomous geometry mapping and robotic navigation in inspection systems. Medium-small (€0.5M - €2M) and medium-large (€2M - €5M) projects represent a substantial portion of the funding landscape. These projects usually aim to develop comprehensive solutions that can be tested and demonstrated in real-world environments. An example is the CompInnova project ^63^, with a budget of €2.5M, which focused on integrating intelligent sensing systems with robotic platforms for automated inspections. Large-scale (above €5M) projects are often collaborative efforts involving multiple stakeholders, including industry leaders, research institutions, and government bodies. These projects aim for significant technological breakthroughs and large-scale implementations. The BugWright2 project (cost of €10M) ^70^ and the RIMA project (cost of €16M) ^71^ exemplify this category, aiming to revolutionise robotic inspection technologies for critical infrastructure through enabling the sharing of best practices, advancing AI and machine learning techniques. Figure 4b and Figure 4c illustrate the impact of the funded projects in the four budget categories in terms of the cumulative number of research outcomes (peer-reviewed journal papers, contribution to conferences and patents). As expected, the number of research outputs increases with the size of the research effort and budget.

### Role of EU regional initiatives

Although the examined case studies cannot represent an exhaustive list, they showcase robotic NDT’s vibrant and innovative landscape in Europe. Each project underscores a commitment to advancing the field through collaborative research, development, and deployment of cutting-edge technologies. These initiatives demonstrate the practical application of robotic NDT in critical sectors and serve as benchmarks for future projects worldwide. It must be noted that the European Commission’s support in robotic NDT and robotic sensing is not fully captured by projects and initiatives directly funded through the HORIZON funding schemes. Regional projects, funded through the European Regional Development Funds (ERDF), have also played a significant role. ERDF is designed to strengthen the EU’s economic, social, and territorial cohesion. The ERDF has financed programmes in shared responsibility between the European Commission and national and regional authorities in Member States. The Member States’ administrations choose which projects to finance and take responsibility for day-to-day management.

An emblematic example is given by the IntACOM programme, part of an initiative known as the Advanced Engineering Materials Research Institute (AEMRI), which is led by TWI’s Advanced Non-destructive Testing Centre (South Wales) and was initially promoted by the Welsh European Funding Office (WEFO) using ERDF. IntACOM pioneered the introduction of robotic phased array ultrasound testing (PAUT) to scan significant components with complex geometries in the aerospace and marine industries. The first IntACOM project started in 2011 and was completed in November 2014. The resulting system delivered a prototype automated inspection cell using two 6-axis robot arms to inspect highly curved components in a fraction of the time usually taken by existing systems ^16^. The latest IntACOM robotic cell can accommodate components up to 12 × 2 × 2m and features an integrated turntable that can accept components up to 4m in diameter and 3m in height. The cell uses CAD data to determine scan paths with the robots working independently or cooperatively to acquire pulse echo or through transmission PAUT data, which is then plotted on 3D images that can be manipulated to provide detailed analysis.

## Regulatory and standardisation efforts

As described in the previous section, under Horizon 2020 and Horizon Europe, the EU has funded research projects that push technological boundaries and contribute to standardisation efforts. Projects such as ROBINS ^68^ and RIMA ^71^ could influence future standards by providing data and findings that feed into the technical committees working on European Norms. As the integration of robotic technologies in NDT and sensing has advanced, the EU has actively pursued regulatory and standardisation initiatives to ensure safety, reliability, and interoperability across the industry. The EU’s commitment to regulatory and standardisation efforts in robotic NDT and sensing is evident in the evolution of relevant European Norms, Directives, and Frameworks. These efforts promote the development of safe and reliable robotic technologies and facilitate a unified market, enabling European entities to lead in the global landscape of robotic NDT and sensing. For robotic NDT, the European Committee for Standardization (CEN) and the European Committee for Electrotechnical Standardization (CENELEC) have been instrumental in developing standards that harmonise methodologies and technical specifications. The EU’s Machinery Directive (2006/42/EC), updated to account for advancements in robotics and AI, establishes a regulatory framework for machinery safety. It encompasses various types of robots, including those used in NDT, by defining essential health and safety requirements. Recent amendments to the Directive aim to address the specific challenges posed by autonomous robots, ensuring that they comply with safety standards and are equipped with necessary safeguards for operation in diverse environments. Industry demands are shifting towards a greater emphasis on sustainability and life-cycle management of assets. This shift will likely increase the demand for robotic NDT systems that can provide thorough and reliable data while minimising waste and the need for costly shutdowns. The role of robotic NDT in predictive maintenance is also expected to grow as industries seek to anticipate failures before they occur. As robotic NDT technologies become more prevalent, the regulatory landscape must adapt. This may include new standards for the certification of robotic inspections and the qualification of systems for specific tasks.

## Challenges, limitations and future trends

Integrating robotics into non-destructive testing (NDT) and sensing offers numerous advantages, yet significant challenges and limitations must be addressed to fully harness these technologies’ potential. The advancement of robotic NDT and sensing technologies presents many challenges and opportunities. Addressing the technical hurdles of complex geometries and material diversity, ensuring scalability and cost-effectiveness, training a skilled workforce, achieving interoperability, and maintaining safety in human-robot collaborative environments are all essential to realising the full potential of these innovations. Overcoming these challenges will require concerted efforts in R&D, education, and regulation, as well as collaboration across industry and academia to develop solutions that are both technologically advanced and practical in real-world applications.

### Technical challenges

One of the principal technical challenges in robotic NDT is the inspection of components with complex geometries. The ability of robotic systems to access and accurately scan intricate surfaces remains limited. While advances in robotic articulation and sensor miniaturisation are ongoing, ensuring complete coverage and reliable data collection over irregular shapes remains challenging. The vast array of materials used in modern manufacturing presents another challenge. Each material can behave differently under testing, requiring a variety of sensors and techniques. Developing a robotic system that can seamlessly switch between methods and calibrate itself for different materials is a complex engineering feat currently at the frontier of R&D. The future of robotic NDT will likely be defined by increased autonomy. Autonomous robotic systems will be capable of performing inspections with minimal human oversight and making decisions about the testing process in real time. Machine learning and AI developments will further enhance these robots’ ability to learn from past inspections, improving their efficiency and accuracy. This progress will enable the inspection of areas currently inaccessible to larger robotic systems, opening new possibilities in NDT applications.

### Scalability of robotic NDT solutions

Scalability is a multifaceted issue in the adoption of robotic NDT solutions. Although robotics can offer increased efficiency and consistency, the initial cost and integration into existing workflows can be significant barriers, especially for small and medium-sized enterprises (SMEs). Furthermore, the customisation required for different inspection scenarios can limit the straightforward scalability of certain robotic NDT technologies.

### Workforce training and safety in human-robot interaction

The shift towards more automated and robotic NDT methods requires a corresponding evolution in workforce skills. Training programs need to be updated to include traditional NDT methods and the operation, maintenance, and troubleshooting of sophisticated robotic systems. Additionally, there is a growing need for data analysts who can interpret the vast amounts of data generated by robotic sensors, transforming it into actionable insights. Accompanying technological progress, European educational institutions and training centres must update curricula and offer specialised courses to prepare a skilled workforce capable of operating and maintaining advanced robotic NDT systems. The development of collaborative robots, or ‘cobots’, designed to work alongside humans, is expected to find a place in NDT. These cobots will be equipped with NDT sensors and will operate in tandem with human inspectors to combine the strengths of both human and robotic capabilities. Introducing robots into the workplace presents new dynamics in human-robot interaction. Ensuring the safety of trained human workers, particularly in environments where robots and humans must coexist, is a critical issue; it involves physical safeguards and sophisticated sensing and avoidance algorithms that can adapt in real-time to the movements of human workers.

### Interoperability and data integration

The principles of Industry 4.0, which include interconnectivity, automation, machine learning, and real-time data, are becoming increasingly integrated with robotic NDT. As different manufacturers often develop robotic systems, there is a lack of standardisation in data formats and communication protocols. That can lead to interoperability issues, where systems or centralised management systems cannot communicate with one another. Ensuring seamless data integration remains a significant hurdle. Future trends will likely lead to more intelligent manufacturing processes and maintenance protocols that leverage the full capabilities of robotic NDT.

## Conclusion

This review has systematically examined the progression and the burgeoning sphere of robotic non-destructive testing (NDT) and sensing within the European context. The critical points discussed have underscored this dynamic field’s technological evolution, current capabilities, and prospective trajectory. We began by tracing the origins of robotic NDT, noting the pivotal milestones that have marked its advancement. The increasing demands for efficiency, precision, and safety in industrial inspections have historically driven the synergy between robotics and NDT techniques. European-led projects and initiatives have been at the forefront of robotic NDT and sensing developments. By leveraging collaborative networks, integrating robotic systems into various industries has been accelerated, enhancing the quality and reliability of inspections. Real-world examples from European projects have demonstrated robotic NDT’s successful application and benefits across sectors, highlighting the value of cross-collaboration between industry, academia, and regulatory bodies. The role of the EU in developing regulatory frameworks and standardisation efforts has been pivotal in ensuring the safety, efficacy, and harmonisation of robotic NDT technologies. The article addressed the technical, scalability, and workforce training challenges currently faced in the sector, noting that while progress has been significant, considerable work remains to achieve ubiquitous integration. With an eye to the future, we discussed upcoming technological advancements and shifts in industry demands. The expected progression towards more autonomous, miniaturised, and intelligent robotic systems is set to transform the landscape of NDT and sensing. Finally, the critical role of collaborations and partnerships has been emphasised. The impact of networks has been significant, fostering environments conducive to innovation and the rapid translation of research into practical applications. In conclusion, the European robotic NDT and sensing landscape is characterised by a rich tapestry of innovation and collaborative efforts. While challenges remain, the concerted efforts by the European Commission, alongside various stakeholders, continue to pave the way for advancements in this field. The strides taken reflect Europe’s position as a global leader in robotic NDT and signal the region’s dedication to embracing the digital transformation of industry. As we look ahead, the continued evolution of robotic NDT and sensing promises to play a critical role in maintaining Europe’s infrastructural integrity, industry competitiveness, and workforce safety.

## Data Availability

The metadata of the projects discussed in this work are available through the following link: https://doi.org/10.5281/zenodo.12205758
