# Supplementary material for: Advancements in integrated robotic sensing: A European perspective
Source: Open Res Eur. 2024 Jul 2;4:39. Originally published 2024 Feb 27. [Version 2] doi: 10.12688/openreseurope.16918.2 (PMC11231631; doi:10.12688/openreseurope.16918.2)
Supplement: Peer review history

# Reviewer report: Reviewer response for version 2

**DOI:** 10.21956/openreseurope.19573.r41845

**Published:** 2024-07-08

**Authors:** Hamidreza Nemati

**Affiliations:** 1 Arizona State University, Tempe, Arizona, USA

The author has revised the manuscript based on the present reviewer's comment. Now, the manuscript is recommended for acceptance in Open Research Europe journal, as it is and being approved for indexing.

Is the review written in accessible language?

Yes

Are all factual statements correct and adequately supported by citations?

Partly

Are the conclusions drawn appropriate in the context of the current research literature?

Yes

Is the topic of the review discussed comprehensively in the context of the current literature?

Yes

Reviewer Expertise:

Smart Structures, Structural Control, None-destructive Evaluation, Bio-inspired Acoustic Sensing, Ultrasound

I confirm that I have read this submission and believe that I have an appropriate level of expertise to confirm that it is of an acceptable scientific standard.

# Reviewer report: Reviewer response for version 1

**DOI:** 10.21956/openreseurope.18284.r40731

**Published:** 2024-06-06

**Authors:** Morteza Tabatabaeipour

**Affiliations:** 1 Ulster University, Coleraine, Northern Ireland, UK

The manuscript titled "Advancements in robotic-enabled sensing: A European perspective" presents a good review of the current state and advancements in robotic Non-Destructive Testing (NDT) and sensing technologies, with a focus on European contributions. The paper is well-organized and covers a wide range of topics, including historical context, current technological advances, European initiatives, and future trends. However, there are some small areas where improvements can be made to enhance the clarity and depth of the discussion.

  

- "Searches were executed across a spectrum of scientific databases, including but not limited to Scopus, Web of Science, IEEE Xplore, and the European Union’s Community Research and Development Information Service (CORDIS)."

What about patents? Robotic NDT advancements may have been carried out with European companies that were not interested in publication but rather in securing patents. Patents need to be further evaluated.

- “For instance, the fusion of visual sensors with acoustic and ultrasonic sensors has enabled the detection of subsurface flaws that are not discernible to the naked eye or traditional imaging.” Any reference for this statement? The role of visual sensors in subsurface detection should be further discussed.

- “Advanced navigation systems, often powered by simultaneous localisation and mapping (SLAM) algorithms, allow robots to understand and navigate their surroundings in real-time.” Since the paper is about robotic NDT, readers expect to see relevant references, for example, for SLAM on NDT. The reference here just relates to advances in robotics.

- “Implementing machine learning algorithms in robotic NDT systems is expected to enable predictive maintenance capabilities. Such systems will not only be able to identify defects but also predict their growth and suggest optimal maintenance schedules.” This is a very general statement, and it is not clear how ML can contribute to predictive maintenance. A relevant robotic NDT reference is also needed.

- The spelling should be American: “Note that the ‘?’ in ‘roboti?ed’ allowed looking for the presence of both the British English word ‘robotised’ and the respective American English version ‘robotised’.”

- A CORDIS database reference should be provided for the readers.

- The sunburst chart of the fields of science (Figure 1a) shows that the CORDIS search resulted in projects relating robotic NDT to social science (sociology). It is expected that the author interpret and discuss this unique case (sociology) in more detail for readers. While readers can understand the relationship between engineering and medical fields, the connection between sociology and NDT is unclear. Economics and business were discussed under the category of social science, but not sociology.

- Any relevant robotic NDT reference for this statement? “The development of collaborative robots, or ‘cobots’, designed to work alongside humans, is expected to find a place in NDT. These cobots will be equipped with NDT sensors and will operate in tandem with human inspectors to combine the strengths of both human and robotic capabilities.”

Thank you for the opportunity to review this manuscript.

Is the review written in accessible language?

Yes

Are all factual statements correct and adequately supported by citations?

Yes

Are the conclusions drawn appropriate in the context of the current research literature?

Yes

Is the topic of the review discussed comprehensively in the context of the current literature?

Yes

Reviewer Expertise:

Ultrasonic Non-destructive Testing and Evaluation (NDT&e), NDT Automation,

I confirm that I have read this submission and believe that I have an appropriate level of expertise to confirm that it is of an acceptable scientific standard.

# Response

**Published:** 2024-06-24

**Authors:** Carmelo Mineo

**Affiliations:** ICAR, National Research Council, Palermo, Italy

Thanks to the reviewer for his valuable comments. Here is how the reviewer's comments were addressed in the revised version of the manuscript.

- "Searches were executed across a spectrum of scientific databases, including but not limited to Scopus, Web of Science, IEEE Xplore, and the European Union’s Community Research and Development Information Service (CORDIS)." What about patents? Robotic NDT advancements may have been carried out with European companies that were not interested in publication but rather in securing patents. Patents need to be further evaluated. 

**Answer **- Thanks to the reviewer for this comment. Additional metadata was extracted from the CORDIS repository to examine information that can be related to the impact produced by the funded projects. For each project, it was found that the repository contains the number of peer-reviewed papers, contributions to conferences, and patents filed. Therefore, Figure 4b and Figure 4c were added to the article. They illustrate the impact of the funded projects in the four budget categories (small, medium-small, medium-large and large-scale) regarding the cumulative number of research outcomes (peer-reviewed journal papers, contribution to conferences and patents). As expected, the number of research outputs increases with the size of the research effort and budget. Unfortunately, the CORDIS repository does not explicitly contain the names of the countries where the patents were filed. Extracting this information would require a lengthy search of every patent produced by each of the 1371 projects considered in this work. Therefore, this was deemed outside the scope of this work.

- “For instance, the fusion of visual sensors with acoustic and ultrasonic sensors has enabled the detection of subsurface flaws that are not discernible to the naked eye or traditional imaging.” Any reference for this statement? The role of visual sensors in subsurface detection should be further discussed.

**Answer **- The following reference was added to the manuscript: Samuelson, C. R., Duffy-Deno, C. A., Whitworth, C. B., Mascareñas, D. D., Tippmann, J. D., & Cattaneo, A. (2020). Visio-Acoustic Data Fusion for Structural Health Monitoring Applications. In Rotating Machinery, Optical Methods & Scanning LDV Methods, Volume 6: Proceedings of the 38th IMAC, A Conference and Exposition on Structural Dynamics 2020 (pp. 37-51). Springer International Publishing. Moreover, the text mentioned by the reviewer was enriched by adding the following statement: “Visual sensors, such as cameras and optical scanners, provide high-resolution images of the surface and near-surface regions. When combined with acoustic or ultrasonic sensors, which penetrate deeper into the material, visual sensors help create a more comprehensive understanding of the inspected object.”

- “Advanced navigation systems, often powered by simultaneous localisation and mapping (SLAM) algorithms, allow robots to understand and navigate their surroundings in realtime.” Since the paper is about robotic NDT, readers expect to see relevant references, for example, for SLAM on NDT. The reference here just relates to advances in robotics.

**Answer** - The following reference was added to the manuscript: Wang, X., Ma, X., & Li, Z. (2023). Research on SLAM and path planning method of inspection robot in complex scenarios. Electronics, 12(10), 2178.

- “Implementing machine learning algorithms in robotic NDT systems is expected to enable predictive maintenance capabilities. Such systems will not only be able to identify defects but also predict their growth and suggest optimal maintenance schedules.” This is a very general statement, and it is not clear how ML can contribute to predictive maintenance. A relevant robotic NDT reference is also needed.

**Answer** - The discussion on Artificial Intelligence, Machine Learning and Deep Learning was extended and supported by the following additional references: Lăzăroiu, G., Andronie, M., Iatagan, M., Geamănu, M., Ștefănescu, R., & Dijmărescu, I. (2022). Deep learning-assisted smart process planning, robotic wireless sensor networks, and geospatial big data management algorithms in the internet of manufacturing things. ISPRS International Journal of Geo-Information, 11(5), 277. Tian, Y., Chen, C., Sagoe-Crentsil, K., Zhang, J., & Duan, W. (2022). Intelligent robotic systems for structural health monitoring: Applications and future trends. Automation in construction, 139, 104273. Davtalab, O., Kazemian, A., Yuan, X., & Khoshnevis, B. (2022). Automated inspection in robotic additive manufacturing using deep learning for layer deformation detection. Journal of Intelligent Manufacturing, 33(3), 771-784. Mena, P., Babicz, G., Banda, S., Kerby, L., & Spirito, C. M. (2023). A Survey of Proposed Standards and Regulations for Artificial Intelligence (No. INL/CON-24-76067-Rev000). Idaho National Laboratory (INL), Idaho Falls, ID (United States).

- The spelling should be American: “Note that the ‘?’ in ‘roboti?ed’ allowed looking for the presence of both the British English word ‘robotised’ and the respective American English version ‘robotised’.”

**Answer **- Thanks to the reviewer for spotting this spelling mistake. It was fixed.

- A CORDIS database reference should be provided for the readers.

**Answer **- Thanks to the reviewer for the comment. The DOI links of all the discussed pivotal projects were included in the bibliography. Additionally, the data extracted from CORDIS to conduct this review were saved into a ZENODO repository and the link was added to the “Data availability” section of the manuscript. The link is this: https://doi.org/10.5281/zenodo.12205758

- The sunburst chart of the fields of science (Figure 1a) shows that the CORDIS search resulted in projects relating robotic NDT to social science (sociology). It is expected that the author interprets and discuss this unique case (sociology) in more detail for readers. While readers can understand the relationship between engineering and medical fields, the connection between sociology and NDT is unclear. Economics and business were discussed under the category of social science, but not sociology.

**Answer** - Thanks to the reviewer for highlighting this. The following text was added to the discussion of the sunburst chart: “‘Sociology’ represents 27.5% of the broad field, which makes it the second major sub-category of ‘Social Sciences.’ The relationship between sociology and NDT requires further exploration. One of the key intersections between sociology and robotic NDT is the study of human-technology interaction. Sociologists investigate how individuals and organizations perceive, adopt, and integrate modern technologies, such as robotic NDT systems, into their practices. This research is essential for understanding the social dynamics that influence the successful implementation of these technologies in various industries. For instance, introducing robotic NDT might necessitate new training programs for workers, leading to shifts in job roles and skill requirements. The role of robotic NDT in ensuring the safety and reliability of critical infrastructures is another area of interest for sociologists. Research in this field explores how different communities perceive the risks associated with infrastructure and the role of robotic NDT in mitigating those risks. Such studies are crucial for developing effective public communication strategies and building trust in innovative technologies. For example, public perception of robotic inspections in the nuclear energy sector can influence policy decisions and industry practices.”

- Any relevant robotic NDT reference for this statement? “The development of collaborative robots, or ‘cobots’, designed to work alongside humans, is expected to find a place in NDT. These cobots will be equipped with NDT sensors and will operate in tandem with human inspectors to combine the strengths of both human and robotic capabilities.”

**Answer** - The following references were added to support the discussion of collaborative robotics in the NDT field: Zimermann, R., Mohseni, E., Vasilev, M., Loukas, C., Vithanage, R. K., Macleod, C. N., ... & Ding, J. (2022). Collaborative robotic wire+ arc additive manufacture and sensor-enabled in-process ultrasonic non-destructive evaluation. Sensors, 22(11), 4203. Buerkle, A., Matharu, H., Al-Yacoub, A., Lohse, N., Bamber, T., & Ferreira, P. (2022). An adaptive human sensor framework for human–robot collaboration. The International Journal of Advanced Manufacturing Technology, 1-16.

# Reviewer report: Reviewer response for version 1

**DOI:** 10.21956/openreseurope.18284.r40239

**Published:** 2024-05-25

**Authors:** Hamidreza Nemati

**Affiliations:** 1 Arizona State University, Tempe, Arizona, USA

The manuscript discusses the advancements in robotic non-destructive testing and sensing, highlighting the significant contributions from European researchers and institutions. It emphasizes the evolution from traditional methods to integrated robotic systems, which enhance precision, speed, efficiency, and safety. The review outlines how these innovations have positioned European researchers at the forefront of the field, influencing global trends and applications across multiple sectors, from aerospace to healthcare, enhancing safety and quality standards worldwide. I think this paper is of a high standard overall, with clearly elaborated discussions and sections. I would approve it after some revisions.

 I have the following comments:

- It is alright to use “robotic-enabled sensing”. however, it is not that common in the literature. I would suggest using terms like “integrated robotic sensing/system” or “robotic sensor integration” instead.
- It is recommended to include a flowchart showing this review's orientation. That way, it would be easier for the readers to follow the article.

- The author discussed the funding/funded projects, but classification based on financial funding/ budget was not discussed. It would be beneficial to highlight this discussion as well.
- As another important indicator to assess the place of NDE research, compared to other fields, it would be better if the percentage of the NDE-based research topic allocated by governmental/federal parties in terms of funding/budget is mentioned and discussed. This would provide a clearer picture of the support and emphasis placed on NDE research in relation to other areas.
- The applicability of the aforementioned projects in industry could be evaluated based on the patents filed. It would be great if the author develop a classification framework that organizes projects based on their patents and the countries where they were filed.
- For a review paper, it is important to reference the most recent studies to ensure the content is up-to-date and relevant. However, many of the cited articles are relatively old. It is recommended to revise the article by adding some recently published papers from 2022 and later.
- As the author mentioned, Recent advances in soft computing, artificial intelligence, machine learning, and deep learning have revolutionized NDE techniques, establishing new research topics. It is recommended that the author provide a comprehensive review of such topics. The author allocated a small portion to this important area in the last paragraph of page 4. It is expected that new proposals in this area are monitored by some standards and codes to guarantee the necessity of conducting such research. To the best knowledge of the reviewer, different NDE organizations around the world deal with this challenge by defining practical research themes filtered by industrial peers. As a result, it is highly recommended that the author include and at least briefly discuss the challenge to address this concern.

Some minor comments:

- Page 6, column 2, end of paragraph 1: Change “robotised” to “robotized” in “the respective American English version “robotised” “.
- Please double-check for grammatical errors throughout the text. For example, on page 8, column 1, end of paragraph 1 change “reflect” to “reflects” in  “as its predominant sub-category (38.8% of the broad field), reflect an intriguing dimension.”
- In the sunburst chart shown in Figure 1(a), page 7, some subsector names are incomplete. Please complete the names in the outer sector.
- Introduce all abbreviations, such as the European Union, at their first appearance in the text. Amend all abbreviations within the manuscript appropriately.

Is the review written in accessible language?

Yes

Are all factual statements correct and adequately supported by citations?

Partly

Are the conclusions drawn appropriate in the context of the current research literature?

Yes

Is the topic of the review discussed comprehensively in the context of the current literature?

Yes

Reviewer Expertise:

Smart Structures, Structural Control, None-destructive Evaluation, Bio-inspired Acoustic Sensing, Ultrasound

I confirm that I have read this submission and believe that I have an appropriate level of expertise to confirm that it is of an acceptable scientific standard, however I have significant reservations, as outlined above.

# Response

**Published:** 2024-06-24

**Authors:** Carmelo Mineo

**Affiliations:** ICAR, National Research Council, Palermo, Italy

Thanks to the reviewer for his valuable comments. Here is how the reviewer's comments were addressed.

**Comment 1** - It is alright to use “robotic-enabled sensing”. however, it is not that common in the literature. I would suggest using terms like “integrated robotic sensing/system” or “robotic sensor integration” instead.

**Answer** - The title was amended, replacing “robotic-enabled sensing” with “integrated robotic sensing”. All other occurrences were modified throughout the article too.

**Comment 2** - It is recommended to include a flowchart showing this review's orientation. That way, it would be easier for the readers to follow the article.

**Answer **- A flowchart was created and inserted into the manuscript. It was placed after the Introduction section, with the following caption: "Figure 1: Flowchart illustrating the structure and key themes of this review article."

**Comment 3** - The author discussed the funding/funded projects, but classification based on financial funding/ budget was not discussed. It would be beneficial to highlight this discussion as well.

**Answer **- A new section titled “Financial classification of funded projects” was added after the introduction of “Pivotal European projects”. This section classifies the projects into four categories based on their total budgets: small, medium-small, medium-large and large-scale. This classification is supported by a new figure (Figure 4).

**Comment 4** - As another important indicator to assess the place of NDE research, compared to other fields, it would be better if the percentage of the NDE-based research topic allocated by governmental/federal parties in terms of funding/budget is mentioned and discussed. This would provide a clearer picture of the support and emphasis placed on NDE research in relation to other areas.

**Answer **- Investigating projects and funding in other fields, with the objective of extracting detailed statistical information, is outside the scope of this work. Nevertheless, the following sentence was added to “The scale of EU-funded initiatives” subsection: “The total cost of the selected projects amounts to over 11 billion euros, with a total EU contribution of 7.3 billion euros (66.2%). Thus, the percentage of other funds is 43.8% of the total. That has most likely been allocated by governmental and federal parties, which provides a clear picture of the support and prioritisation of robotic sensing research within the broader research landscape.”

**Comment 5** - The applicability of the aforementioned projects in industry could be evaluated based on the patents filed. It would be great if the author develop a classification framework that organises projects based on their patents and the countries where they were filed. **Answer** - Thanks to the reviewer for this comment. Additional metadata was extracted from the CORDIS repository to examine information that can be related to the impact produced by the funded projects. For each project, it was found that the repository contains the number of peer-reviewed papers, contributions to conferences, and patents filed. Therefore, Figure 4b and Figure 4c were added to the article. They illustrate the impact of the funded projects in the four budget categories (small, medium-small, medium-large and large-scale) regarding the cumulative number of research outcomes (peer-reviewed journal papers, contribution to conferences and patents). As expected, the number of research outputs increases with the size of the research effort and budget. Unfortunately, the CORDIS repository does not explicitly contain the names of the countries where the patents were filed. Extracting this information would require a lengthy search of every patent produced by each of the 1371 projects considered in this work. Therefore, this was deemed outside the scope of this work.

**Comment 6** - For a review paper, it is important to reference the most recent studies to ensure the content is up-to-date and relevant. However, many of the cited articles are relatively old. It is recommended to revise the article by adding some recently published papers from 2022 and later.

**Answer** - Thanks to the reviewer for his comment. The following recent papers were identified and reviewed and incorporated into the manuscript:

- Lăzăroiu, G., Andronie, M., Iatagan, M., Geamănu, M., Ștefănescu, R., & Dijmărescu, I. (2022). Deep learning-assisted smart process planning, robotic wireless sensor networks, and geospatial big data management algorithms in the internet of manufacturing things. ISPRS International Journal of Geo-Information, 11(5), 277.
- Best, G., Garg, R., Keller, J., Hollinger, G. A., & Scherer, S. (2024). Multi-robot, multi-sensor exploration of multifarious environments with full mission aerial autonomy. The International Journal of Robotics Research, 43(4), 485-512.
- Verma, A., Kaiwart, A., Dubey, N. D., Naseer, F., & Pradhan, S. (2022). A review on various types of in-pipe inspection robot. Materials Today: Proceedings, 50, 1425-1434.
- Liu, Y., Hajj, M., & Bao, Y. (2022). Review of robot-based damage assessment for offshore wind turbines. Renewable and Sustainable Energy Reviews, 158, 112187.
- Tian, Y., Chen, C., Sagoe-Crentsil, K., Zhang, J., & Duan, W. (2022). Intelligent robotic systems for structural health monitoring: Applications and future trends. Automation in construction, 139, 104273.
- Davtalab, O., Kazemian, A., Yuan, X., & Khoshnevis, B. (2022). Automated inspection in robotic additive manufacturing using deep learning for layer deformation detection. Journal of Intelligent Manufacturing, 33(3), 771-784.
- Wang, X., Ma, X., & Li, Z. (2023). Research on SLAM and path planning method of inspection robot in complex scenarios. Electronics, 12(10), 2178.
- Buerkle, A., Matharu, H., Al-Yacoub, A., Lohse, N., Bamber, T., & Ferreira, P. (2022). An adaptive human sensor framework for human–robot collaboration. The International Journal of Advanced Manufacturing Technology, 1-16.
- Zimermann, R., Mohseni, E., Vasilev, M., Loukas, C., Vithanage, R. K., Macleod, C. N., ... & Ding, J. (2022). Collaborative robotic wire+ arc additive manufacture and sensor-enabled in-process ultrasonic non-destructive evaluation. Sensors, 22(11), 4203.
- Mena, P., Babicz, G., Banda, S., Kerby, L., & Spirito, C. M. (2023). A Survey of Proposed Standards and Regulations for Artificial Intelligence (No. INL/CON-24-76067-Rev000). Idaho National Laboratory (INL), Idaho Falls, ID (United States).

**Comment 7 **- As the author mentioned, Recent advances in soft computing, artificial intelligence, machine learning, and deep learning have revolutionised NDE techniques, establishing new research topics. It is recommended that the author provide a comprehensive review of such topics. The author allocated a small portion to this important area in the last paragraph of page 4. It is expected that new proposals in this area are monitored by some standards and codes to guarantee the necessity of conducting such research. To the best knowledge of the reviewer, different NDE organisations around the world deal with this challenge by defining practical research themes filtered by industrial peers. As a result, it is highly recommended that the author include and at least briefly discuss the challenge to address this concern.

**Answer** - The following text was added to the manuscript: “Soft computing techniques, including fuzzy logic, genetic algorithms, and neural networks, are increasingly utilized in NDE for tasks such as signal processing, pattern recognition, and decision making. These methods offer robustness and flexibility, accommodating the uncertainties and variabilities inherent in NDE data. AI and ML algorithms are deployed to automate the analysis of complex NDE data, identifying patterns and anomalies that may be imperceptible to human inspectors. For example, convolutional neural networks (CNNs) and support vector machines (SVMs) are used for image-based inspection tasks, such as defect detection in welds and composite materials. Deep learning, a subset of ML, leverages multi-layered neural networks to model intricate relationships within data. In NDE, DL techniques are employed for tasks such as ultrasonic signal classification, radiographic image interpretation, and predictive maintenance. These approaches improve detection accuracy and reduce false positives, enabling the interpretation of vast datasets with improved accuracy and the ability to learn from historical inspection data to optimise future inspections, thereby enhancing the reliability of NDE processes. To ensure the necessity and quality of research in this area, various standards and codes are being established. Organizations such as the American Society for Non-destructive Testing (ASNT), the International Organization for Standardization (ISO), and the European Federation for Non-Destructive Testing (EFNDT) are providing guidelines and standards for the implementation of AI, ML, and DL in NDE. These standards ensure that new technologies are rigorously tested and validated before being applied in industrial settings [40]”.

**Minor comments:**

- Page 6, column 2, end of paragraph 1: Change “robotised” to “robotised” in “the respective American English version “robotised” “.

Answer: Done.

- Please double-check for grammatical errors throughout the text. For example, on page 8, column 1, end of paragraph 1 change “reflect” to “reflects” in  “as its predominant sub-category (38.8% of the broad field), reflect an intriguing dimension.

Answer: Done.

- In the sunburst chart shown in Figure 1(a), page 7, some subsector names are incomplete. Please complete the names in the outer sector.

Answer: The sunburst chart was improved as recommended.

- Introduce all abbreviations, such as the European Union, at their first appearance in the text. Amend all abbreviations within the manuscript appropriately.

Answer: Done.
